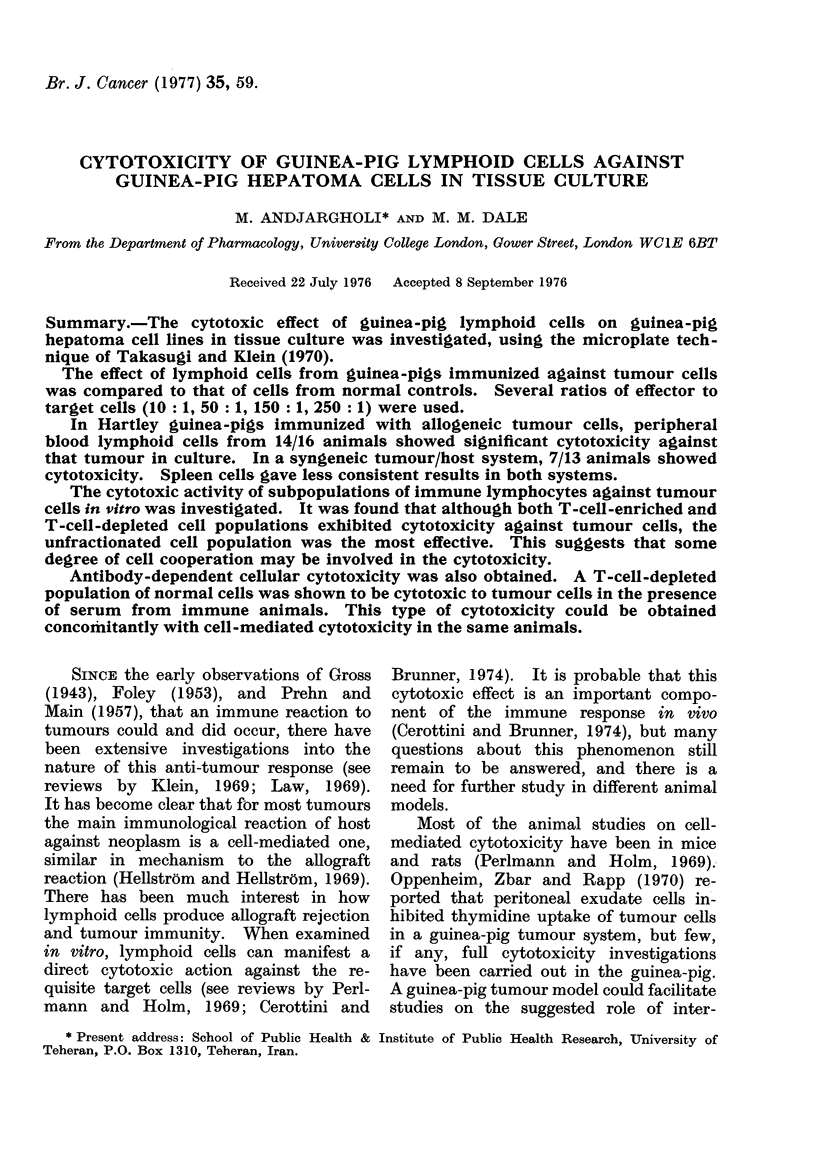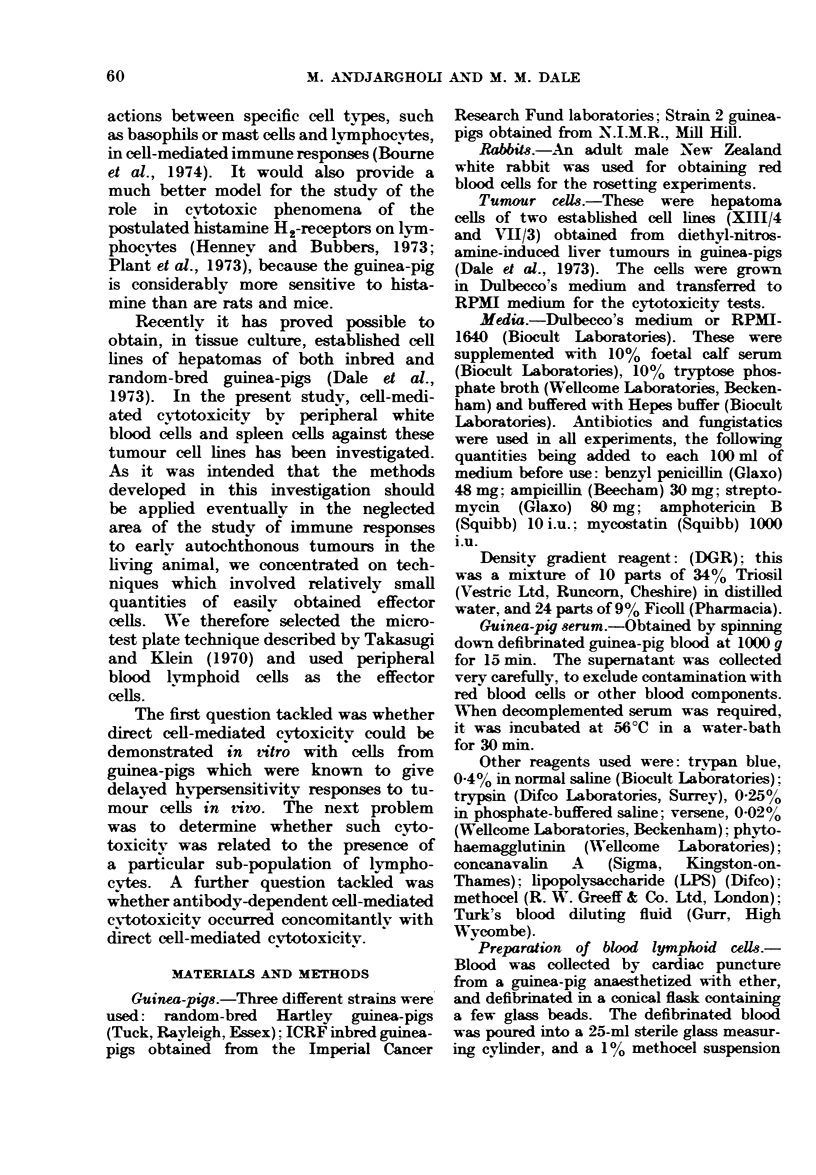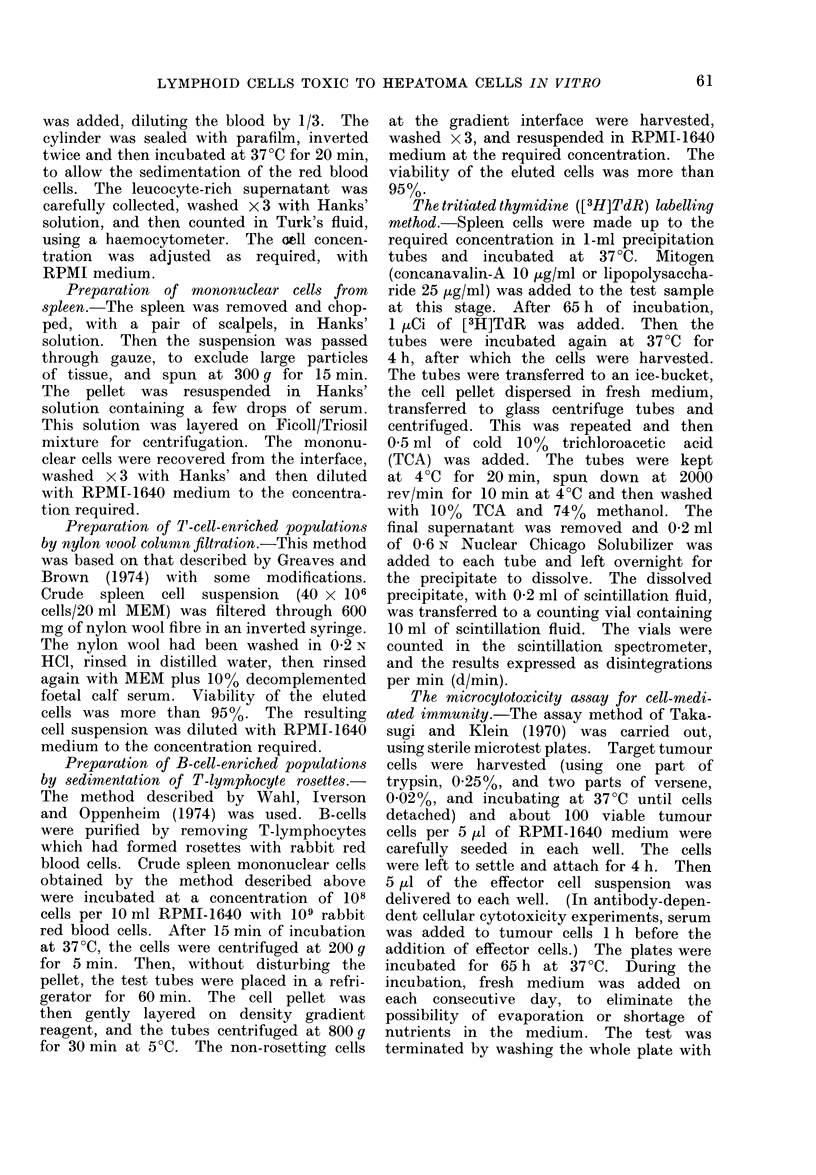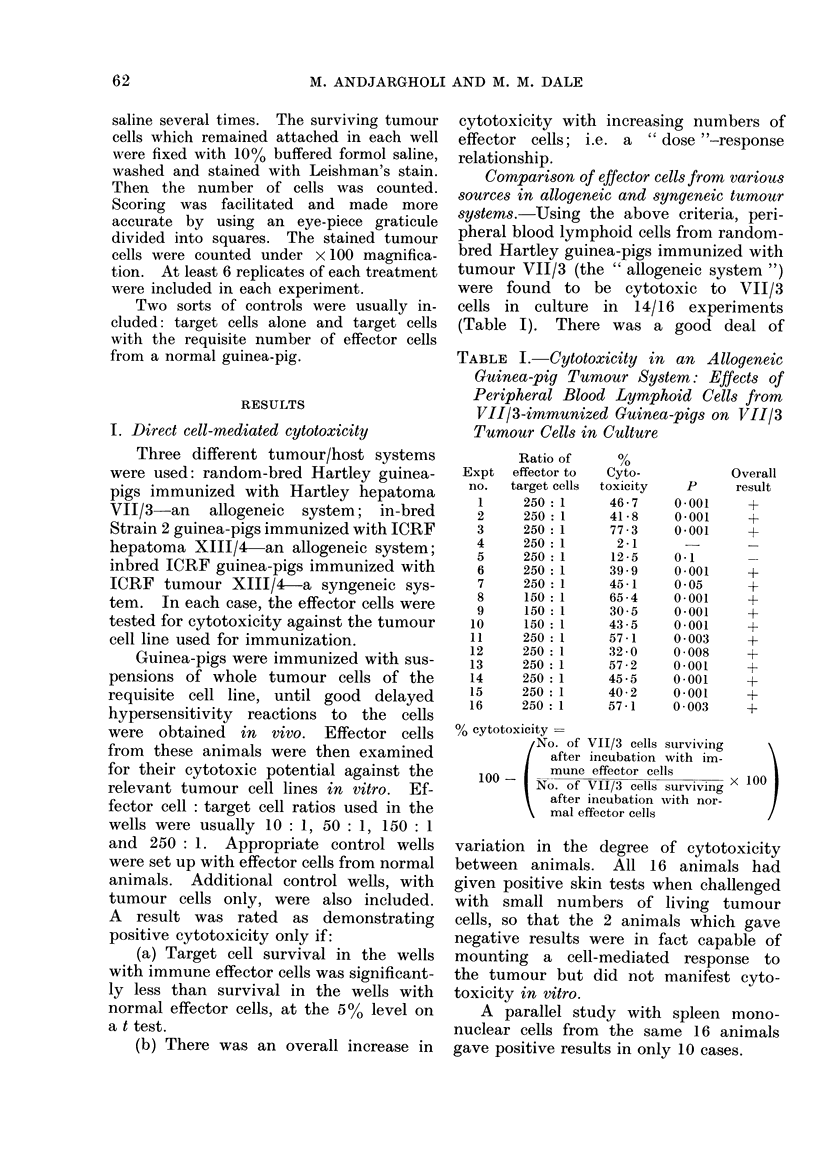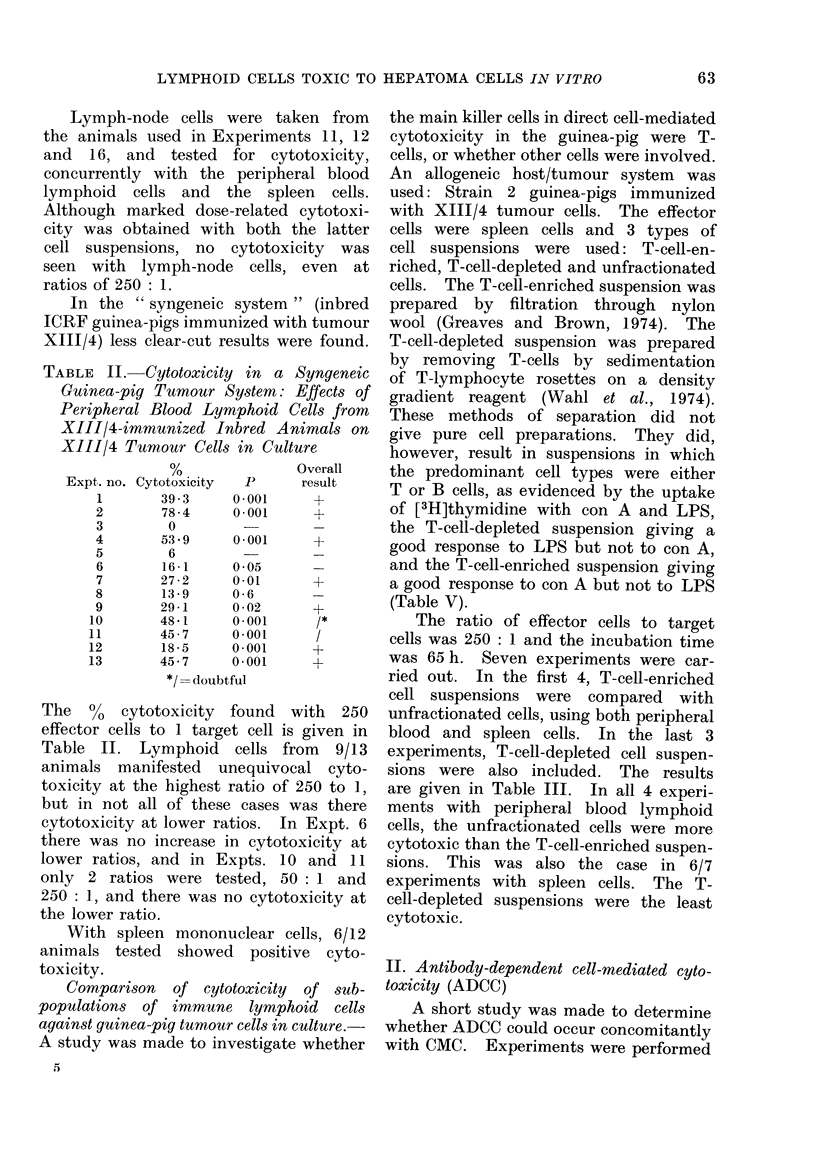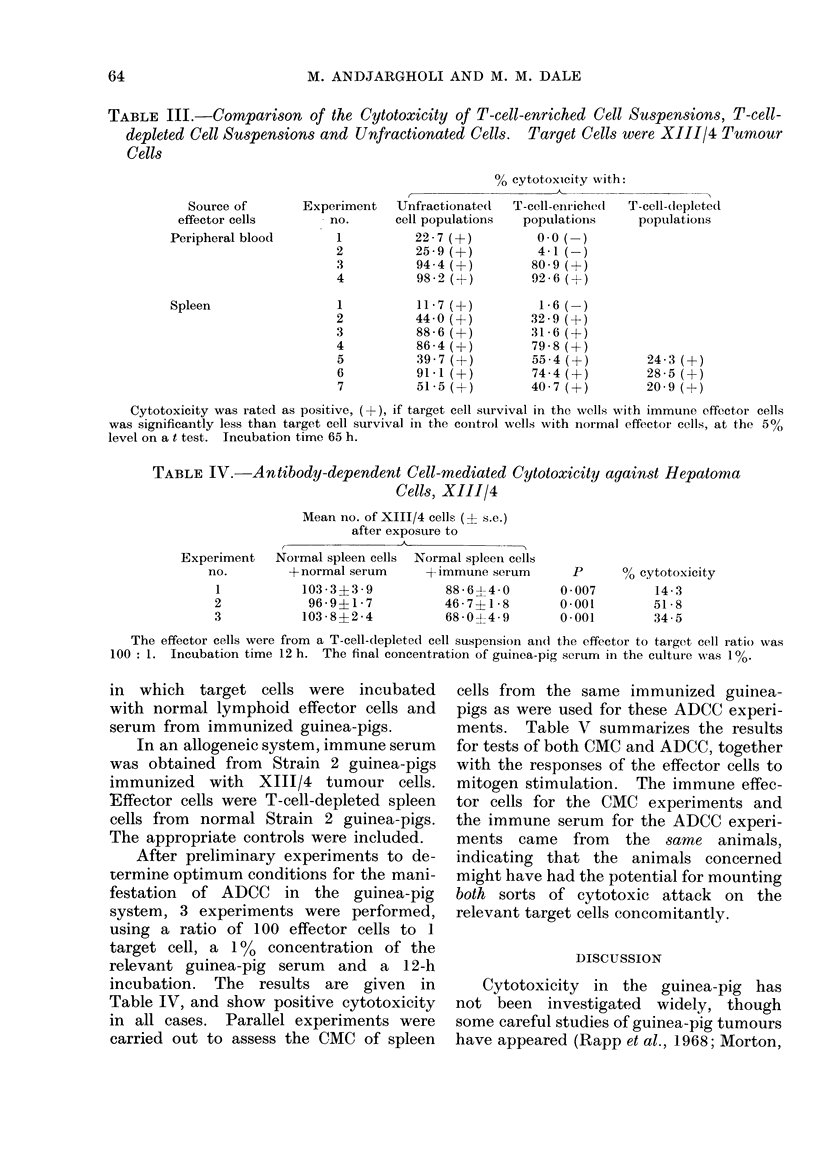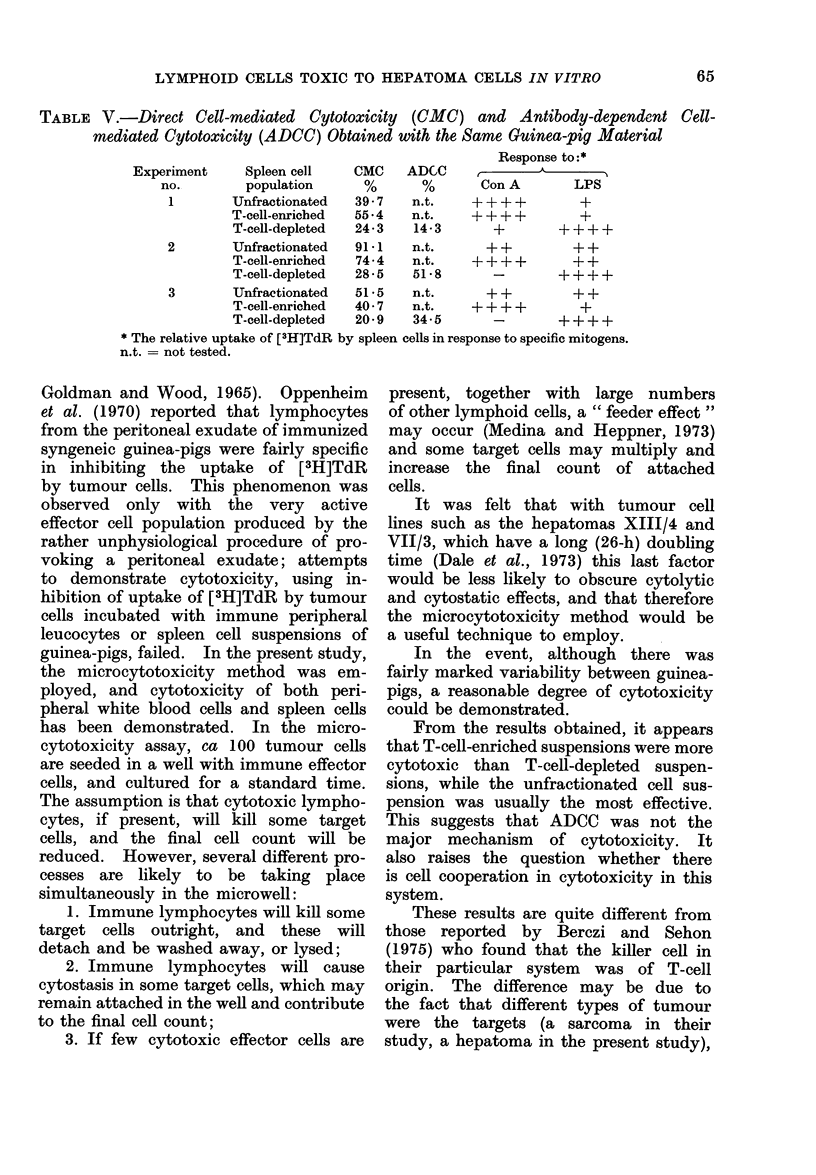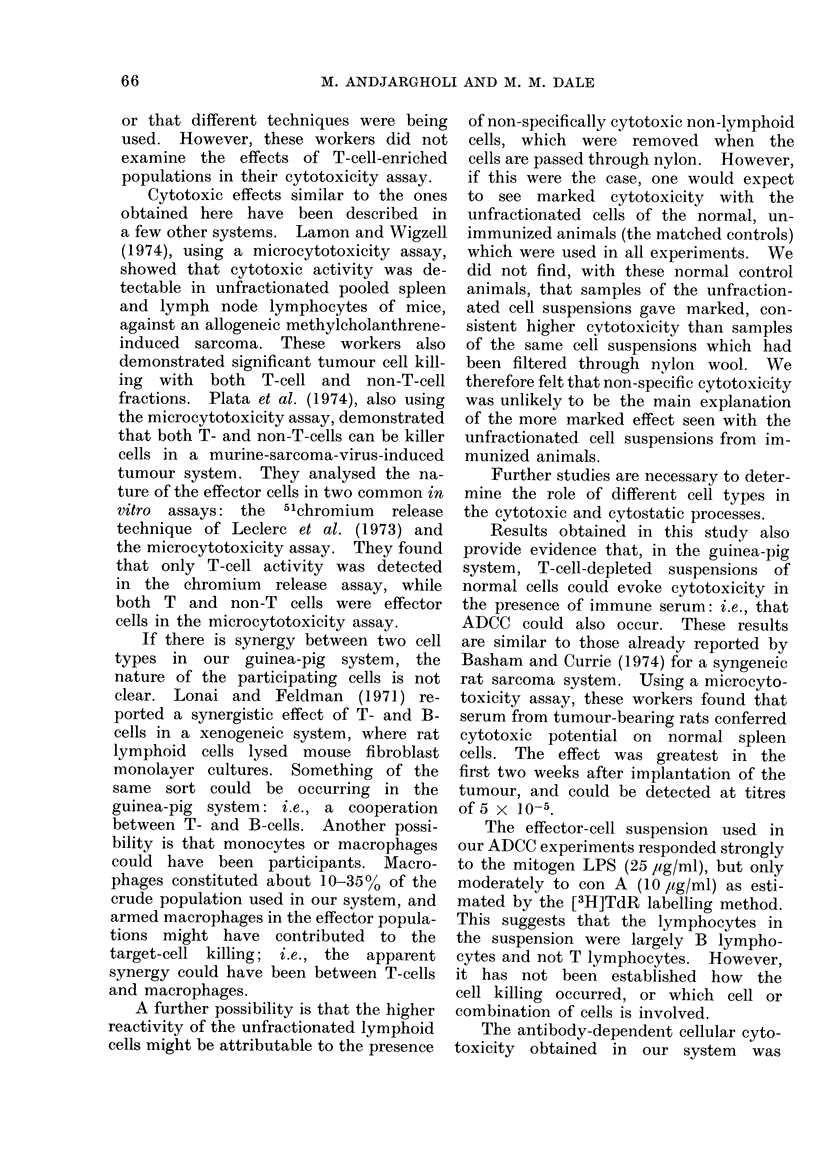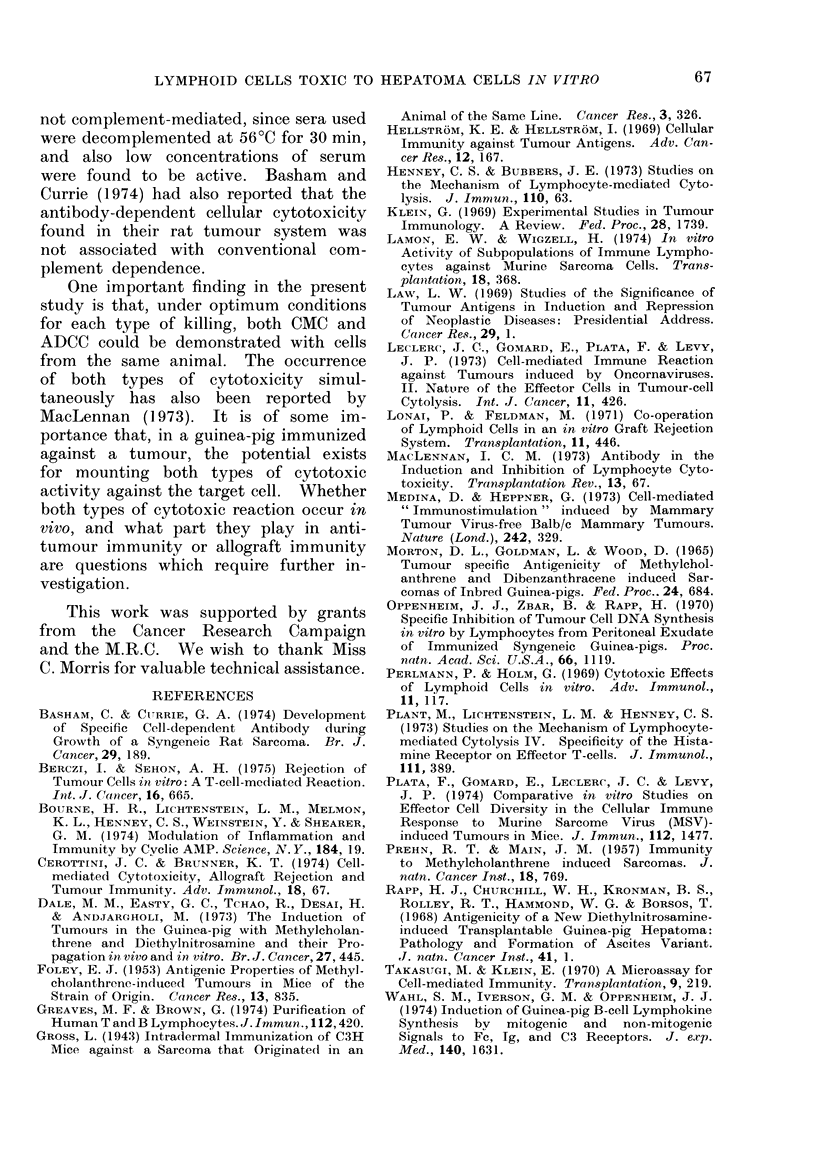# Cytotoxicity of guinea-pig lymphoid cells against guinea-pig hepatoma cells in tissue culture.

**DOI:** 10.1038/bjc.1977.4

**Published:** 1977-01

**Authors:** M. Andjargholi, M. M. Dale

## Abstract

The cytotoxic effect of guinea-pig lymphoid cells on guinea-pig hepatoma cell lines in tissue culture was investigated, using the microplate technique of Takasugi and Klein (1970). The effect of lymphoid cells from guinea-pigs immunized against tumor cells was compared to that of cells from normal controls. Several ratios of effector to target cells (10 : 1, 50 : 1, 150: 1, 250 : 1) were used. In Hartley guinea-pigs immunized with allogeneic tumour cells, peripheral blood lymphoid cells from 14/16 animals showed significant cytotoxicity against that tumour in culture. In a syngeneic tumour/host system, 7/13 animals showed cytotoxicity. Spleen cells gave less consistent results in both systems. The cytotoxic activity of subpopulations of immune lymphocytes against tumour cells in vitro was investigated. It was found that although both T-cell-enriched and T-cell-depleted cell populations exhibited cytotoxicity against tumour cells, the unfractionated cell population was the most effective. This suggests that some degree of cell cooperation may be involved in the cytotoxicity. Antibody-dependent cellular cytotoxicity was also obtained. A T-cell-depleted population of normal cells was shown to be cytotoxic to tumour cells in the presence of serum from immune animals. This type of cytotoxicity could be obtained concomitantly with cell-mediated cytotoxicity in the same animals.


*Br. J. Cancer* (1977) **35**, 59.

### CYTOTOXICITY OF GUINEA-PIG LYMPHOID CELLS AGAINST GUINEA-PIG HEPATOMA CELLS IN TISSUE CULTURE

M. ANDJARGHOLI* AND M. M. DALE

*From the Department of Pharmacology, University College London, Gower Street, London WC1E 6BT*

Received 22 July 1976 Accepted 8 September 1976

**Summary.—The cytotoxic effect of guinea-pig lymphoid cells on guinea-pig hepatoma cell lines in tissue culture was investigated, using the microplate technique of Takasugi and Klein (1970).**

**The effect of lymphoid cells from guinea-pigs immunized against tumour cells was compared to that of cells from normal controls. Several ratios of effector to target cells (10 : 1, 50 : 1, 150 : 1, 250 : 1) were used.**

**In Hartley guinea-pigs immunized with allogeneic tumour cells, peripheral blood lymphoid cells from 14/16 animals showed significant cytotoxicity against that tumour in culture. In a syngeneic tumour/host system, 7/13 animals showed cytotoxicity. Spleen cells gave less consistent results in both systems.**

**The cytotoxic activity of subpopulations of immune lymphocytes against tumour cells *in vitro* was investigated. It was found that although both T-cell-enriched and T-cell-depleted cell populations exhibited cytotoxicity against tumour cells, the unfractionated cell population was the most effective. This suggests that some degree of cell cooperation may be involved in the cytotoxicity.**

**Antibody-dependent cellular cytotoxicity was also obtained. A T-cell-depleted population of normal cells was shown to be cytotoxic to tumour cells in the presence of serum from immune animals. This type of cytotoxicity could be obtained concomitantly with cell-mediated cytotoxicity in the same animals.**

SINCE the early observations of Gross (1943), Foley (1953), and Prehn and Main (1957), that an immune reaction to tumours could and did occur, there have been extensive investigations into the nature of this anti-tumour response (see reviews by Klein, 1969; Law, 1969). It has become clear that for most tumours the main immunological reaction of host against neoplasm is a cell-mediated one, similar in mechanism to the allograft reaction (Hellström and Hellström, 1969). There has been much interest in how lymphoid cells produce allograft rejection and tumour immunity. When examined *in vitro*, lymphoid cells can manifest a direct cytotoxic action against the requisite target cells (see reviews by Perlmann and Holm, 1969; Cerottini and Brunner, 1974). It is probable that this cytotoxic effect is an important component of the immune response *in vivo* (Cerottini and Brunner, 1974), but many questions about this phenomenon still remain to be answered, and there is a need for further study in different animal models.

Most of the animal studies on cell-mediated cytotoxicity have been in mice and rats (Perlmann and Holm, 1969). Oppenheim, Zbar and Rapp (1970) reported that peritoneal exudate cells inhibited thymidine uptake of tumour cells in a guinea-pig tumour system, but few, if any, full cytotoxicity investigations have been carried out in the guinea-pig. A guinea-pig tumour model could facilitate studies on the suggested role of inter-

* Present address: School of Public Health & Institute of Public Health Research, University of Teheran, P.O. Box 1310, Teheran, Iran.

actions between specific cell types, such as basophils or mast cells and lymphocytes, in cell-mediated immune responses (Bourne *et al.*, 1974). It would also provide a much better model for the study of the role in cytotoxic phenomena of the postulated histamine $H_2$-receptors on lymphocytes (Henney and Bubbers, 1973; Plant *et al.*, 1973), because the guinea-pig is considerably more sensitive to histamine than are rats and mice.

Recently it has proved possible to obtain, in tissue culture, established cell lines of hepatomas of both inbred and random-bred guinea-pigs (Dale *et al.*, 1973). In the present study, cell-mediated cytotoxicity by peripheral white blood cells and spleen cells against these tumour cell lines has been investigated. As it was intended that the methods developed in this investigation should be applied eventually in the neglected area of the study of immune responses to early autochthonous tumours in the living animal, we concentrated on techniques which involved relatively small quantities of easily obtained effector cells. We therefore selected the microtest plate technique described by Takasugi and Klein (1970) and used peripheral blood lymphoid cells as the effector cells.

The first question tackled was whether direct cell-mediated cytoxicity could be demonstrated *in vitro* with cells from guinea-pigs which were known to give delayed hypersensitivity responses to tumour cells *in vivo*. The next problem was to determine whether such cytotoxicity was related to the presence of a particular sub-population of lymphocytes. A further question tackled was whether antibody-dependent cell-mediated cytotoxicity occurred concomitantly with direct cell-mediated cytotoxicity.

### MATERIALS AND METHODS

*Guinea-pigs.*—Three different strains were used: random-bred Hartley guinea-pigs (Tuck, Rayleigh, Essex); ICRF inbred guinea-pigs obtained from the Imperial Cancer Research Fund laboratories; Strain 2 guinea-pigs obtained from N.I.M.R., Mill Hill.

*Rabbits.*—An adult male New Zealand white rabbit was used for obtaining red blood cells for the rosetting experiments.

*Tumour cells.*—These were hepatoma cells of two established cell lines (XIII/4 and VII/3) obtained from diethyl-nitrosamine-induced liver tumours in guinea-pigs (Dale *et al.*, 1973). The cells were grown in Dulbecco's medium and transferred to RPMI medium for the cytotoxicity tests.

*Media.*—Dulbecco's medium or RPMI-1640 (Biocult Laboratories). These were supplemented with 10% foetal calf serum (Biocult Laboratories), 10% tryptose phosphate broth (Wellcome Laboratories, Beckenham) and buffered with Hepes buffer (Biocult Laboratories). Antibiotics and fungistatics were used in all experiments, the following quantities being added to each 100 ml of medium before use: benzyl penicillin (Glaxo) 48 mg; ampicillin (Beecham) 30 mg; streptomycin (Glaxo) 80 mg; amphotericin B (Squibb) 10 i.u.; mycostatin (Squibb) 1000 i.u.

Density gradient reagent: (DGR); this was a mixture of 10 parts of 34% Triosil (Vestric Ltd, Runcorn, Cheshire) in distilled water, and 24 parts of 9% Ficoll (Pharmacia).

*Guinea-pig serum.*—Obtained by spinning down defibrinated guinea-pig blood at 1000 *g* for 15 min. The supernatant was collected very carefully, to exclude contamination with red blood cells or other blood components. When decomplemented serum was required, it was incubated at 56°C in a water-bath for 30 min.

Other reagents used were: trypan blue, 0·4% in normal saline (Biocult Laboratories); trypsin (Difco Laboratories, Surrey), 0·25% in phosphate-buffered saline; versene, 0·02% (Wellcome Laboratories, Beckenham); phytohaemagglutinin (Wellcome Laboratories); concanavalin A (Sigma, Kingston-on-Thames); lipopolysaccharide (LPS) (Difco); methocel (R. W. Greeff & Co. Ltd, London); Turk's blood diluting fluid (Gurr, High Wycombe).

*Preparation of blood lymphoid cells.*—Blood was collected by cardiac puncture from a guinea-pig anaesthetized with ether, and defibrinated in a conical flask containing a few glass beads. The defibrinated blood was poured into a 25-ml sterile glass measuring cylinder, and a 1% methocel suspension was added, diluting the blood by 1/3. The cylinder was sealed with parafilm, inverted twice and then incubated at 37 °C for 20 min, to allow the sedimentation of the red blood cells. The leucocyte-rich supernatant was carefully collected, washed ×3 with Hanks' solution, and then counted in Turk's fluid, using a haemocytometer. The cell concentration was adjusted as required, with RPMI medium.

*Preparation of mononuclear cells from spleen.*—The spleen was removed and chopped, with a pair of scalpels, in Hanks' solution. Then the suspension was passed through gauze, to exclude large particles of tissue, and spun at 300 *g* for 15 min. The pellet was resuspended in Hanks' solution containing a few drops of serum. This solution was layered on Ficoll/Triosil mixture for centrifugation. The mononuclear cells were recovered from the interface, washed ×3 with Hanks' and then diluted with RPMI-1640 medium to the concentration required.

*Preparation of T-cell-enriched populations by nylon wool column filtration.*—This method was based on that described by Greaves and Brown (1974) with some modifications. Crude spleen cell suspension ($40 \times 10^6$ cells/20 ml MEM) was filtered through 600 mg of nylon wool fibre in an inverted syringe. The nylon wool had been washed in 0·2 N HCl, rinsed in distilled water, then rinsed again with MEM plus 10% decomplemented foetal calf serum. Viability of the eluted cells was more than 95%. The resulting cell suspension was diluted with RPMI-1640 medium to the concentration required.

*Preparation of B-cell-enriched populations by sedimentation of T-lymphocyte rosettes.*—The method described by Wahl, Iverson and Oppenheim (1974) was used. B-cells were purified by removing T-lymphocytes which had formed rosettes with rabbit red blood cells. Crude spleen mononuclear cells obtained by the method described above were incubated at a concentration of $10^8$ cells per 10 ml RPMI-1640 with $10^9$ rabbit red blood cells. After 15 min of incubation at 37 °C, the cells were centrifuged at 200 *g* for 5 min. Then, without disturbing the pellet, the test tubes were placed in a refrigerator for 60 min. The cell pellet was then gently layered on density gradient reagent, and the tubes centrifuged at 800 *g* for 30 min at 5 °C. The non-rosetting cells at the gradient interface were harvested, washed ×3, and resuspended in RPMI-1640 medium at the required concentration. The viability of the eluted cells was more than 95%.

*The tritiated thymidine* ([$^3H$]*TdR*) *labelling method.*—Spleen cells were made up to the required concentration in 1-ml precipitation tubes and incubated at 37 °C. Mitogen (concanavalin-A 10 μg/ml or lipopolysaccharide 25 μg/ml) was added to the test sample at this stage. After 65 h of incubation, 1 μCi of [$^3$H]TdR was added. Then the tubes were incubated again at 37 °C for 4 h, after which the cells were harvested. The tubes were transferred to an ice-bucket, the cell pellet dispersed in fresh medium, transferred to glass centrifuge tubes and centrifuged. This was repeated and then 0·5 ml of cold 10% trichloroacetic acid (TCA) was added. The tubes were kept at 4 °C for 20 min, spun down at 2000 rev/min for 10 min at 4 °C and then washed with 10% TCA and 74% methanol. The final supernatant was removed and 0·2 ml of 0·6 N Nuclear Chicago Solubilizer was added to each tube and left overnight for the precipitate to dissolve. The dissolved precipitate, with 0·2 ml of scintillation fluid, was transferred to a counting vial containing 10 ml of scintillation fluid. The vials were counted in the scintillation spectrometer, and the results expressed as disintegrations per min (d/min).

*The microcytotoxicity assay for cell-mediated immunity.*—The assay method of Takasugi and Klein (1970) was carried out, using sterile microtest plates. Target tumour cells were harvested (using one part of trypsin, 0·25%, and two parts of versene, 0·02%, and incubating at 37 °C until cells detached) and about 100 viable tumour cells per 5 μl of RPMI-1640 medium were carefully seeded in each well. The cells were left to settle and attach for 4 h. Then 5 μl of the effector cell suspension was delivered to each well. (In antibody-dependent cellular cytotoxicity experiments, serum was added to tumour cells 1 h before the addition of effector cells.) The plates were incubated for 65 h at 37 °C. During the incubation, fresh medium was added on each consecutive day, to eliminate the possibility of evaporation or shortage of nutrients in the medium. The test was terminated by washing the whole plate with saline several times. The surviving tumour cells which remained attached in each well were fixed with 10% buffered formol saline, washed and stained with Leishman's stain. Then the number of cells was counted. Scoring was facilitated and made more accurate by using an eye-piece graticule divided into squares. The stained tumour cells were counted under ×100 magnification. At least 6 replicates of each treatment were included in each experiment.

Two sorts of controls were usually included: target cells alone and target cells with the requisite number of effector cells from a normal guinea-pig.

### RESULTS

### I. *Direct cell-mediated cytotoxicity*

Three different tumour/host systems were used: random-bred Hartley guinea-pigs immunized with Hartley hepatoma VII/3—an allogeneic system; in-bred Strain 2 guinea-pigs immunized with ICRF hepatoma XIII/4—an allogeneic system; inbred ICRF guinea-pigs immunized with ICRF tumour XIII/4—a syngeneic system. In each case, the effector cells were tested for cytotoxicity against the tumour cell line used for immunization.

Guinea-pigs were immunized with suspensions of whole tumour cells of the requisite cell line, until good delayed hypersensitivity reactions to the cells were obtained *in vivo*. Effector cells from these animals were then examined for their cytotoxic potential against the relevant tumour cell lines *in vitro*. Effector cell : target cell ratios used in the wells were usually 10 : 1, 50 : 1, 150 : 1 and 250 : 1. Appropriate control wells were set up with effector cells from normal animals. Additional control wells, with tumour cells only, were also included. A result was rated as demonstrating positive cytotoxicity only if:

(a) Target cell survival in the wells with immune effector cells was significantly less than survival in the wells with normal effector cells, at the 5% level on a *t* test.

(b) There was an overall increase in cytotoxicity with increasing numbers of effector cells; i.e. a " dose "–response relationship.

*Comparison of effector cells from various sources in allogeneic and syngeneic tumour systems.*—Using the above criteria, peripheral blood lymphoid cells from random-bred Hartley guinea-pigs immunized with tumour VII/3 (the " allogeneic system ") were found to be cytotoxic to VII/3 cells in culture in 14/16 experiments (Table I). There was a good deal of variation in the degree of cytotoxicity between animals. All 16 animals had given positive skin tests when challenged with small numbers of living tumour cells, so that the 2 animals which gave negative results were in fact capable of mounting a cell-mediated response to the tumour but did not manifest cytotoxicity *in vitro*.

A parallel study with spleen mononuclear cells from the same 16 animals gave positive results in only 10 cases.

TABLE I.—*Cytotoxicity in an Allogeneic Guinea-pig Tumour System: Effects of Peripheral Blood Lymphoid Cells from VII/3-immunized Guinea-pigs on VII/3 Tumour Cells in Culture*

| Expt no. | Ratio of effector to target cells | % Cyto-toxicity | *P* | Overall result |
|---|---|---|---|---|
| 1 | 250 : 1 | 46·7 | 0·001 | + |
| 2 | 250 : 1 | 41·8 | 0·001 | + |
| 3 | 250 : 1 | 77·3 | 0·001 | + |
| 4 | 250 : 1 | 2·1 | — | − |
| 5 | 250 : 1 | 12·5 | 0·1 | − |
| 6 | 250 : 1 | 39·9 | 0·001 | + |
| 7 | 250 : 1 | 45·1 | 0·05 | + |
| 8 | 150 : 1 | 65·4 | 0·001 | + |
| 9 | 150 : 1 | 30·5 | 0·001 | + |
| 10 | 150 : 1 | 43·5 | 0·001 | + |
| 11 | 250 : 1 | 57·1 | 0·003 | + |
| 12 | 250 : 1 | 32·0 | 0·008 | + |
| 13 | 250 : 1 | 57·2 | 0·001 | + |
| 14 | 250 : 1 | 45·5 | 0·001 | + |
| 15 | 250 : 1 | 40·2 | 0·001 | + |
| 16 | 250 : 1 | 57·1 | 0·003 | + |

$$\%\ \text{cytotoxicity} = 100 - \left(\frac{\text{No. of VII/3 cells surviving after incubation with immune effector cells}}{\text{No. of VII/3 cells surviving after incubation with normal effector cells}} \times 100\right)$$

Lymph-node cells were taken from the animals used in Experiments 11, 12 and 16, and tested for cytotoxicity, concurrently with the peripheral blood lymphoid cells and the spleen cells. Although marked dose-related cytotoxicity was obtained with both the latter cell suspensions, no cytotoxicity was seen with lymph-node cells, even at ratios of 250 : 1.

In the " syngeneic system " (inbred ICRF guinea-pigs immunized with tumour XIII/4) less clear-cut results were found.

TABLE II.—*Cytotoxicity in a Syngeneic Guinea-pig Tumour System: Effects of Peripheral Blood Lymphoid Cells from XIII/4-immunized Inbred Animals on XIII/4 Tumour Cells in Culture*

| Expt. no. | % Cytotoxicity | $P$ | Overall result |
|---|---|---|---|
| 1 | 39·3 | 0·001 | + |
| 2 | 78·4 | 0·001 | + |
| 3 | 0 | — | — |
| 4 | 53·9 | 0·001 | + |
| 5 | 6 | — | — |
| 6 | 16·1 | 0·05 | — |
| 7 | 27·2 | 0·01 | + |
| 8 | 13·9 | 0·6 | — |
| 9 | 29·1 | 0·02 | + |
| 10 | 48·1 | 0·001 | /* |
| 11 | 45·7 | 0·001 | / |
| 12 | 18·5 | 0·001 | + |
| 13 | 45·7 | 0·001 | + |

*/ = doubtful

The % cytotoxicity found with 250 effector cells to 1 target cell is given in Table II. Lymphoid cells from 9/13 animals manifested unequivocal cytotoxicity at the highest ratio of 250 to 1, but in not all of these cases was there cytotoxicity at lower ratios. In Expt. 6 there was no increase in cytotoxicity at lower ratios, and in Expts. 10 and 11 only 2 ratios were tested, 50 : 1 and 250 : 1, and there was no cytotoxicity at the lower ratio.

With spleen mononuclear cells, 6/12 animals tested showed positive cytotoxicity.

*Comparison of cytotoxicity of subpopulations of immune lymphoid cells against guinea-pig tumour cells in culture.*—A study was made to investigate whether the main killer cells in direct cell-mediated cytotoxicity in the guinea-pig were T-cells, or whether other cells were involved. An allogeneic host/tumour system was used: Strain 2 guinea-pigs immunized with XIII/4 tumour cells. The effector cells were spleen cells and 3 types of cell suspensions were used: T-cell-enriched, T-cell-depleted and unfractionated cells. The T-cell-enriched suspension was prepared by filtration through nylon wool (Greaves and Brown, 1974). The T-cell-depleted suspension was prepared by removing T-cells by sedimentation of T-lymphocyte rosettes on a density gradient reagent (Wahl *et al.*, 1974). These methods of separation did not give pure cell preparations. They did, however, result in suspensions in which the predominant cell types were either T or B cells, as evidenced by the uptake of [$^3$H]thymidine with con A and LPS, the T-cell-depleted suspension giving a good response to LPS but not to con A, and the T-cell-enriched suspension giving a good response to con A but not to LPS (Table V).

The ratio of effector cells to target cells was 250 : 1 and the incubation time was 65 h. Seven experiments were carried out. In the first 4, T-cell-enriched cell suspensions were compared with unfractionated cells, using both peripheral blood and spleen cells. In the last 3 experiments, T-cell-depleted cell suspensions were also included. The results are given in Table III. In all 4 experiments with peripheral blood lymphoid cells, the unfractionated cells were more cytotoxic than the T-cell-enriched suspensions. This was also the case in 6/7 experiments with spleen cells. The T-cell-depleted suspensions were the least cytotoxic.

II. *Antibody-dependent cell-mediated cytotoxicity* (ADCC)

A short study was made to determine whether ADCC could occur concomitantly with CMC. Experiments were performed

TABLE III.—*Comparison of the Cytotoxicity of T-cell-enriched Cell Suspensions, T-cell-depleted Cell Suspensions and Unfractionated Cells. Target Cells were XIII/4 Tumour Cells*

| Source of effector cells | Experiment no. | % cytotoxicity with: Unfractionated cell populations | T-cell-enriched populations | T-cell-depleted populations |
|---|---|---|---|---|
| Peripheral blood | 1 | 22·7 (+) | 0·0 (−) | |
| | 2 | 25·9 (+) | 4·1 (−) | |
| | 3 | 94·4 (+) | 80·9 (+) | |
| | 4 | 98·2 (+) | 92·6 (+) | |
| Spleen | 1 | 11·7 (+) | 1·6 (−) | |
| | 2 | 44·0 (+) | 32·9 (+) | |
| | 3 | 88·6 (+) | 31·6 (+) | |
| | 4 | 86·4 (+) | 79·8 (+) | |
| | 5 | 39·7 (+) | 55·4 (+) | 24·3 (+) |
| | 6 | 91·1 (+) | 74·4 (+) | 28·5 (+) |
| | 7 | 51·5 (+) | 40·7 (+) | 20·9 (+) |

Cytotoxicity was rated as positive, (+), if target cell survival in the wells with immune effector cells was significantly less than target cell survival in the control wells with normal effector cells, at the 5% level on a *t* test. Incubation time 65 h.

TABLE IV.—*Antibody-dependent Cell-mediated Cytotoxicity against Hepatoma Cells, XIII/4*

| Experiment no. | Mean no. of XIII/4 cells (± s.e.) after exposure to: Normal spleen cells + normal serum | Normal spleen cells + immune serum | *P* | % cytotoxicity |
|---|---|---|---|---|
| 1 | 103·3±3·9 | 88·6±4·0 | 0·007 | 14·3 |
| 2 | 96·9±1·7 | 46·7±1·8 | 0·001 | 51·8 |
| 3 | 103·8±2·4 | 68·0±4·9 | 0·001 | 34·5 |

The effector cells were from a T-cell-depleted cell suspension and the effector to target cell ratio was 100 : 1. Incubation time 12 h. The final concentration of guinea-pig serum in the culture was 1%.

in which target cells were incubated with normal lymphoid effector cells and serum from immunized guinea-pigs.

In an allogeneic system, immune serum was obtained from Strain 2 guinea-pigs immunized with XIII/4 tumour cells. Effector cells were T-cell-depleted spleen cells from normal Strain 2 guinea-pigs. The appropriate controls were included.

After preliminary experiments to determine optimum conditions for the manifestation of ADCC in the guinea-pig system, 3 experiments were performed, using a ratio of 100 effector cells to 1 target cell, a 1% concentration of the relevant guinea-pig serum and a 12-h incubation. The results are given in Table IV, and show positive cytotoxicity in all cases. Parallel experiments were carried out to assess the CMC of spleen cells from the same immunized guinea-pigs as were used for these ADCC experiments. Table V summarizes the results for tests of both CMC and ADCC, together with the responses of the effector cells to mitogen stimulation. The immune effector cells for the CMC experiments and the immune serum for the ADCC experiments came from the *same* animals, indicating that the animals concerned might have had the potential for mounting *both* sorts of cytotoxic attack on the relevant target cells concomitantly.

### DISCUSSION

Cytotoxicity in the guinea-pig has not been investigated widely, though some careful studies of guinea-pig tumours have appeared (Rapp *et al.*, 1968; Morton,

TABLE V.—*Direct Cell-mediated Cytotoxicity (CMC) and Antibody-dependent Cell-mediated Cytotoxicity (ADCC) Obtained with the Same Guinea-pig Material*

| Experiment no. | Spleen cell population | CMC % | ADCC % | Response to:* Con A | LPS |
|---|---|---|---|---|---|
| 1 | Unfractionated | 39·7 | n.t. | ++++ | + |
| | T-cell-enriched | 55·4 | n.t. | ++++ | + |
| | T-cell-depleted | 24·3 | 14·3 | + | ++++ |
| 2 | Unfractionated | 91·1 | n.t. | ++ | ++ |
| | T-cell-enriched | 74·4 | n.t. | ++++ | ++ |
| | T-cell-depleted | 28·5 | 51·8 | – | ++++ |
| 3 | Unfractionated | 51·5 | n.t. | ++ | ++ |
| | T-cell-enriched | 40·7 | n.t. | ++++ | + |
| | T-cell-depleted | 20·9 | 34·5 | – | ++++ |

* The relative uptake of [$^3$H]TdR by spleen cells in response to specific mitogens.
n.t. = not tested.

Goldman and Wood, 1965). Oppenheim *et al.* (1970) reported that lymphocytes from the peritoneal exudate of immunized syngeneic guinea-pigs were fairly specific in inhibiting the uptake of [$^3$H]TdR by tumour cells. This phenomenon was observed only with the very active effector cell population produced by the rather unphysiological procedure of provoking a peritoneal exudate; attempts to demonstrate cytotoxicity, using inhibition of uptake of [$^3$H]TdR by tumour cells incubated with immune peripheral leucocytes or spleen cell suspensions of guinea-pigs, failed. In the present study, the microcytotoxicity method was employed, and cytotoxicity of both peripheral white blood cells and spleen cells has been demonstrated. In the microcytotoxicity assay, *ca* 100 tumour cells are seeded in a well with immune effector cells, and cultured for a standard time. The assumption is that cytotoxic lymphocytes, if present, will kill some target cells, and the final cell count will be reduced. However, several different processes are likely to be taking place simultaneously in the microwell:

1. Immune lymphocytes will kill some target cells outright, and these will detach and be washed away, or lysed;

2. Immune lymphocytes will cause cytostasis in some target cells, which may remain attached in the well and contribute to the final cell count;

3. If few cytotoxic effector cells are present, together with large numbers of other lymphoid cells, a " feeder effect " may occur (Medina and Heppner, 1973) and some target cells may multiply and increase the final count of attached cells.

It was felt that with tumour cell lines such as the hepatomas XIII/4 and VII/3, which have a long (26-h) doubling time (Dale *et al.*, 1973) this last factor would be less likely to obscure cytolytic and cytostatic effects, and that therefore the microcytotoxicity method would be a useful technique to employ.

In the event, although there was fairly marked variability between guinea-pigs, a reasonable degree of cytotoxicity could be demonstrated.

From the results obtained, it appears that T-cell-enriched suspensions were more cytotoxic than T-cell-depleted suspensions, while the unfractionated cell suspension was usually the most effective. This suggests that ADCC was not the major mechanism of cytotoxicity. It also raises the question whether there is cell cooperation in cytotoxicity in this system.

These results are quite different from those reported by Berczi and Sehon (1975) who found that the killer cell in their particular system was of T-cell origin. The difference may be due to the fact that different types of tumour were the targets (a sarcoma in their study, a hepatoma in the present study), or that different techniques were being used. However, these workers did not examine the effects of T-cell-enriched populations in their cytotoxicity assay.

Cytotoxic effects similar to the ones obtained here have been described in a few other systems. Lamon and Wigzell (1974), using a microcytotoxicity assay, showed that cytotoxic activity was detectable in unfractionated pooled spleen and lymph node lymphocytes of mice, against an allogeneic methylcholanthrene-induced sarcoma. These workers also demonstrated significant tumour cell killing with both T-cell and non-T-cell fractions. Plata *et al.* (1974), also using the microcytotoxicity assay, demonstrated that both T- and non-T-cells can be killer cells in a murine-sarcoma-virus-induced tumour system. They analysed the nature of the effector cells in two common *in vitro* assays: the $^{51}$chromium release technique of Leclerc *et al.* (1973) and the microcytotoxicity assay. They found that only T-cell activity was detected in the chromium release assay, while both T and non-T cells were effector cells in the microcytotoxicity assay.

If there is synergy between two cell types in our guinea-pig system, the nature of the participating cells is not clear. Lonai and Feldman (1971) reported a synergistic effect of T- and B-cells in a xenogeneic system, where rat lymphoid cells lysed mouse fibroblast monolayer cultures. Something of the same sort could be occurring in the guinea-pig system: *i.e.*, a cooperation between T- and B-cells. Another possibility is that monocytes or macrophages could have been participants. Macrophages constituted about 10–35% of the crude population used in our system, and armed macrophages in the effector populations might have contributed to the target-cell killing; *i.e.*, the apparent synergy could have been between T-cells and macrophages.

A further possibility is that the higher reactivity of the unfractionated lymphoid cells might be attributable to the presence of non-specifically cytotoxic non-lymphoid cells, which were removed when the cells are passed through nylon. However, if this were the case, one would expect to see marked cytotoxicity with the unfractionated cells of the normal, unimmunized animals (the matched controls) which were used in all experiments. We did not find, with these normal control animals, that samples of the unfractionated cell suspensions gave marked, consistent higher cytotoxicity than samples of the same cell suspensions which had been filtered through nylon wool. We therefore felt that non-specific cytotoxicity was unlikely to be the main explanation of the more marked effect seen with the unfractionated cell suspensions from immunized animals.

Further studies are necessary to determine the role of different cell types in the cytotoxic and cytostatic processes.

Results obtained in this study also provide evidence that, in the guinea-pig system, T-cell-depleted suspensions of normal cells could evoke cytotoxicity in the presence of immune serum: *i.e.*, that ADCC could also occur. These results are similar to those already reported by Basham and Currie (1974) for a syngeneic rat sarcoma system. Using a microcytotoxicity assay, these workers found that serum from tumour-bearing rats conferred cytotoxic potential on normal spleen cells. The effect was greatest in the first two weeks after implantation of the tumour, and could be detected at titres of $5 \times 10^{-5}$.

The effector-cell suspension used in our ADCC experiments responded strongly to the mitogen LPS (25 $\mu$g/ml), but only moderately to con A (10 $\mu$g/ml) as estimated by the [$^3$H]TdR labelling method. This suggests that the lymphocytes in the suspension were largely B lymphocytes and not T lymphocytes. However, it has not been established how the cell killing occurred, or which cell or combination of cells is involved.

The antibody-dependent cellular cytotoxicity obtained in our system was not complement-mediated, since sera used were decomplemented at 56°C for 30 min, and also low concentrations of serum were found to be active. Basham and Currie (1974) had also reported that the antibody-dependent cellular cytotoxicity found in their rat tumour system was not associated with conventional complement dependence.

One important finding in the present study is that, under optimum conditions for each type of killing, both CMC and ADCC could be demonstrated with cells from the same animal. The occurrence of both types of cytotoxicity simultaneously has also been reported by MacLennan (1973). It is of some importance that, in a guinea-pig immunized against a tumour, the potential exists for mounting both types of cytotoxic activity against the target cell. Whether both types of cytotoxic reaction occur *in vivo*, and what part they play in anti-tumour immunity or allograft immunity are questions which require further investigation.

This work was supported by grants from the Cancer Research Campaign and the M.R.C. We wish to thank Miss C. Morris for valuable technical assistance.

REFERENCES

BASHAM, C. & CURRIE, G. A. (1974) Development of Specific Cell-dependent Antibody during Growth of a Syngeneic Rat Sarcoma. *Br. J. Cancer*, **29**, 189.

BERCZI, I. & SEHON, A. H. (1975) Rejection of Tumour Cells *in vitro*: A T-cell-mediated Reaction. *Int. J. Cancer*, **16**, 665.

BOURNE, H. R., LICHTENSTEIN, L. M., MELMON, K. L., HENNEY, C. S., WEINSTEIN, Y. & SHEARER, G. M. (1974) Modulation of Inflammation and Immunity by Cyclic AMP. *Science, N.Y.*, **184**, 19.

CEROTTINI, J. C. & BRUNNER, K. T. (1974) Cell-mediated Cytotoxicity, Allograft Rejection and Tumour Immunity. *Adv. Immunol.*, **18**, 67.

DALE, M. M., EASTY, G. C., TCHAO, R., DESAI, H. & ANDJARGHOLI, M. (1973) The Induction of Tumours in the Guinea-pig with Methylcholanthrene and Diethylnitrosamine and their Propagation *in vivo* and *in vitro*. *Br. J. Cancer*, **27**, 445.

FOLEY, E. J. (1953) Antigenic Properties of Methylcholanthrene-induced Tumours in Mice of the Strain of Origin. *Cancer Res.*, **13**, 835.

GREAVES, M. F. & BROWN, G. (1974) Purification of Human T and B Lymphocytes. *J. Immun.*, **112**, 420.

GROSS, L. (1943) Intradermal Immunization of C3H Mice against a Sarcoma that Originated in an Animal of the Same Line. *Cancer Res.*, **3**, 326.

HELLSTRÖM, K. E. & HELLSTRÖM, I. (1969) Cellular Immunity against Tumour Antigens. *Adv. Cancer Res.*, **12**, 167.

HENNEY, C. S. & BUBBERS, J. E. (1973) Studies on the Mechanism of Lymphocyte-mediated Cytolysis. *J. Immun.*, **110**, 63.

KLEIN, G. (1969) Experimental Studies in Tumour Immunology. A Review. *Fed. Proc.*, **28**, 1739.

LAMON, E. W. & WIGZELL, H. (1974) *In vitro* Activity of Subpopulations of Immune Lymphocytes against Murine Sarcoma Cells. *Transplantation*, **18**, 368.

LAW, L. W. (1969) Studies of the Significance of Tumour Antigens in Induction and Repression of Neoplastic Diseases: Presidential Address. *Cancer Res.*, **29**, 1.

LECLERC, J. C., GOMARD, E., PLATA, F. & LEVY, J. P. (1973) Cell-mediated Immune Reaction against Tumours induced by Oncornaviruses. II. Nature of the Effector Cells in Tumour-cell Cytolysis. *Int. J. Cancer*, **11**, 426.

LONAI, P. & FELDMAN, M. (1971) Co-operation of Lymphoid Cells in an *in vitro* Graft Rejection System. *Transplantation*, **11**, 446.

MACLENNAN, I. C. M. (1973) Antibody in the Induction and Inhibition of Lymphocyte Cytotoxicity. *Transplantation Rev.*, **13**, 67.

MEDINA, D. & HEPPNER, G. (1973) Cell-mediated "Immunostimulation" induced by Mammary Tumour Virus-free Balb/c Mammary Tumours. *Nature (Lond.)*, **242**, 329.

MORTON, D. L., GOLDMAN, L. & WOOD, D. (1965) Tumour specific Antigenicity of Methylcholanthrene and Dibenzanthracene induced Sarcomas of Inbred Guinea-pigs. *Fed. Proc.*, **24**, 684.

OPPENHEIM, J. J., ZBAR, B. & RAPP, H. (1970) Specific Inhibition of Tumour Cell DNA Synthesis *in vitro* by Lymphocytes from Peritoneal Exudate of Immunized Syngeneic Guinea-pigs. *Proc. natn. Acad. Sci. U.S.A.*, **66**, 1119.

PERLMANN, P. & HOLM, G. (1969) Cytotoxic Effects of Lymphoid Cells *in vitro*. *Adv. Immunol.*, **11**, 117.

PLANT, M., LICHTENSTEIN, L. M. & HENNEY, C. S. (1973) Studies on the Mechanism of Lymphocyte-mediated Cytolysis IV. Specificity of the Histamine Receptor on Effector T-cells. *J. Immunol.*, **111**, 389.

PLATA, F., GOMARD, E., LECLERC, J. C. & LEVY, J. P. (1974) Comparative *in vitro* Studies on Effector Cell Diversity in the Cellular Immune Response to Murine Sarcome Virus (MSV)-induced Tumours in Mice. *J. Immun.*, **112**, 1477.

PREHN, R. T. & MAIN, J. M. (1957) Immunity to Methylcholanthrene induced Sarcomas. *J. natn. Cancer Inst.*, **18**, 769.

RAPP, H. J., CHURCHILL, W. H., KRONMAN, B. S., ROLLEY, R. T., HAMMOND, W. G. & BORSOS, T. (1968) Antigenicity of a New Diethylnitrosamine-induced Transplantable Guinea-pig Hepatoma: Pathology and Formation of Ascites Variant. *J. natn. Cancer Inst.*, **41**, 1.

TAKASUGI, M. & KLEIN, E. (1970) A Microassay for Cell-mediated Immunity. *Transplantation*, **9**, 219.

WAHL, S. M., IVERSON, G. M. & OPPENHEIM, J. J. (1974) Induction of Guinea-pig B-cell Lymphokine Synthesis by mitogenic and non-mitogenic Signals to Fc, Ig, and C3 Receptors. *J. exp. Med.*, **140**, 1631.